# Tetra­aqua­(nitrato-κ^2^ *O*,*O*′)bis­(pyridinium-4-carboxyl­ate-κ*O*)europium(III) dinitrate

**DOI:** 10.1107/S1600536811014772

**Published:** 2011-04-29

**Authors:** Zhi-Guo Zhong, Jin-Fan Song, Jing Li, Zhao-Hui Meng

**Affiliations:** a Center of Analysis and Testing, Nanyang Normal University, Nanyang 473061, People’s Republic of China; b School of Physics and Electronic Engineering, Nanyang Normal University, Nanyang 473061, People’s Republic of China; c School of Mathematics and Statistics, Nanyang Normal University, Nanyang 473061, People’s Republic of China; d College of Chemistry and Pharmacy Engineering, Nanyang Normal University, Nanyang 473061, People’s Republic of China

## Abstract

The asymmetric unit of the title compound, [Eu(NO_3_)(C_6_H_5_NO_2_)_2_(H_2_O)_4_](NO_3_)_2_, consists of one-half of the *C*_2_ symmetric coordination cation and one nitrate anion. The eight-coordinated Eu^III^ atom is in a distorted dodeca­hedral coordination environment. The coordination cations and nitrate anions are connected *via* O—H⋯O and N—H⋯O hydrogen bonds into a three-dimensional network.

## Related literature

For photophysical properties of lanthanide(III) coordination compounds, see, for example: Jüstel *et al.* (1998 ▶); Xu *et al.* (2010 ▶). For potential applications of lanthanide(III) coordination compounds as light-conversion mol­ecular devices, see, for example: Lehn (1990 ▶).
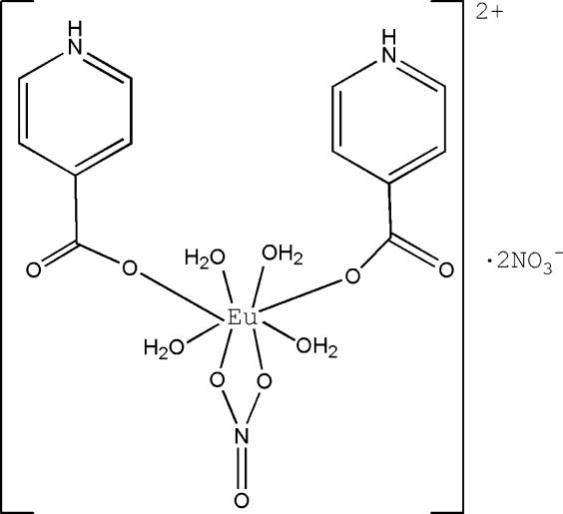


## Experimental

### 

#### Crystal data

[Eu(NO_3_)(C_6_H_5_NO_2_)_2_(H_2_O)_4_](NO_3_)_2_
*M* *_r_* = 656.27
Monoclinic, 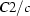

*a* = 14.612 (4) Å
*b* = 12.498 (4) Å
*c* = 13.342 (4) Å
β = 118.728 (4)°
*V* = 2136.6 (11) Å^3^
*Z* = 4
Mo *K*α radiation
μ = 3.03 mm^−1^
*T* = 293 K
0.35 × 0.32 × 0.28 mm

#### Data collection

Bruker APEXII CCD diffractometer
Absorption correction: multi-scan *SADABS* (Bruker, 1997 ▶) *T*_min_ = 0.417, *T*_max_ = 0.484
5224 measured reflections
1881 independent reflections
1847 reflections with *I* > 2σ(*I*)
*R*_int_ = 0.017

#### Refinement

*R*[*F* ^2^ > 2σ(*F* ^2^)] = 0.015
*wR*(*F* ^2^) = 0.038
*S* = 1.02
1881 reflections
180 parameters
8 restraints
H atoms treated by a mixture of independent and constrained refinement
Δρ_max_ = 0.58 e Å^−3^
Δρ_min_ = −0.62 e Å^−3^

### 

Data collection: *SMART* (Bruker, 1997 ▶); cell refinement: *SAINT* (Bruker, 1997 ▶); data reduction: *SAINT*; program(s) used to solve structure: *SHELXS97* (Sheldrick, 2008 ▶); program(s) used to refine structure: *SHELXL97* (Sheldrick, 2008 ▶); molecular graphics: *SHELXTL* (Sheldrick, 2008 ▶); software used to prepare material for publication: *SHELXTL*.

## Supplementary Material

Crystal structure: contains datablocks I, global. DOI: 10.1107/S1600536811014772/gk2366sup1.cif

Structure factors: contains datablocks I. DOI: 10.1107/S1600536811014772/gk2366Isup2.hkl

Additional supplementary materials:  crystallographic information; 3D view; checkCIF report

## Figures and Tables

**Table 1:** Hydrogen-bond geometry (Å, °)

*D*—H⋯*A*	*D*—H	H⋯*A*	*D*⋯*A*	*D*—H⋯*A*
O1*W*—H1*WB*⋯O7^i^	0.81 (2)	1.99 (2)	2.790 (2)	175 (3)
O1*W*—H1*WA*⋯O1^ii^	0.83 (2)	1.87 (2)	2.653 (2)	155 (2)
O2*W*—H2*WB*⋯O1^iii^	0.83 (2)	1.84 (2)	2.661 (2)	173 (3)
O2*W*—H2*WA*⋯O5	0.82 (2)	2.23 (2)	2.958 (3)	148 (3)
N1—H6⋯O7^iv^	0.94 (3)	1.88 (3)	2.814 (3)	179 (3)

Symmetry codes: (i) 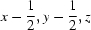; (ii) 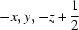; (iii) 
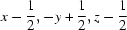
; (iv) 
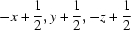
.

**Fig. 1.**
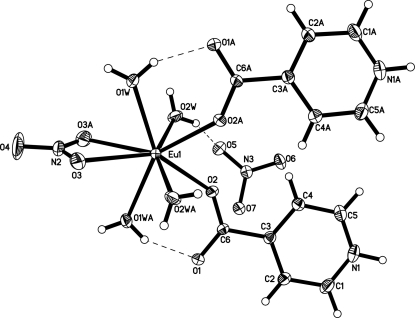
View of the title compound with displacement ellipsoids drawn at the 30% probability level . Symmetry code for the atoms with the label A: -x, y, -z + 1/2.

**Fig. 2.**
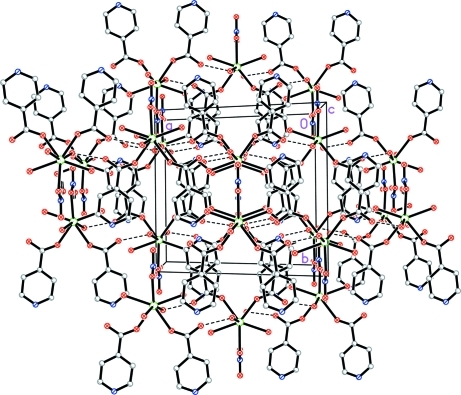
Crystal packing viewed along the c axis. Hydrogen bonds are shown with dashed lines.

## supplementary crystallographic information

### Comment

The lanthanide (III) coordination compounds have attracted considerable attention due to their interesting photophysical properties and their potential application as light-conversion molecular devices. Herein, we report a new rare-earth metal–organic compound [Eu(C_6_H_5_NO_2_)_2_(H_2_O)_4_(NO_3_)](NO_3_)_2_. The structural unit of the title compound consists of one coordination cation which has a crystallographic twofold axis symmetry, [Eu(C_6_H_5_NO_2_)_2_(H_2_O)_4_(NO_3_)]^2+^, and two nitrate anions (Fig. 1). In the coordination cation the Eu(III) center is coordinated by eight O atoms: two from C_6_H_5_NO_2_ ligands, two from NO_3_^- ^anion and four from water molecules. The coordination geometry around the Eu(III) center can be described as dodecahedral with O—Eu—O bond angles ranging from 51.07 (10) to 152.96 (9)° and the Eu—O bond lengths ranging from 2.3614 (16) to 2.5000 (19) Å. The electrostatic interactions and hydrogen bonds result in the formation of three-dimensional network (Fig. 2). Obviously, electrostatic interactions and hydrogen bonds play a crucial role in the chemical stability of the title compound.

### Experimental

All chemicals were of reagent grade quality obtained from commercial sources and used without further purification. 0.24 g of isonicotinic acid (1 mmol) and 0.45 g Eu(NO_3_)_3_.6H_2_O (1 mmol) were dissolved in 30 ml of distilled water. Then pH of the mixture was carefully adjusted to 5.0 with 1*M* HCl solution. After stirring for half an hour, the solution was filtered and left for slowly evaporation at room temperature to obtain colorless crystals suitable for X-ray structure determination.

### Refinement

The H atoms bonded to C were positioned geometrically and refined using a riding model, with C—H = 0.93 Å and with *U*_iso_(H) = 1.2 times *U*_eq_(C). The H atoms bonded to O atoms were located from Fourier difference maps and refined with distance restraints of O1W—H1WA = 0.82, O1W—H1WB = 0.82, O2W—H2WA = 0.82, O2W—H2WB = 0.82, H1WA···H1WB = 1.36, H2WA···H2WB = 1.36 and H1WA···Eu1 = 2.85 Å. The H atom bonded to N atom was located from Fourier difference maps and freely refined. In addition, the O4 and N2 atoms were refined with SHELXL97 restraint 'DELUdelu 0.01'.

### Crystal data

**Table:**

[Eu(NO_3_)(C_6_H_5_NO_2_)_2_(H_2_O)_4_](NO_3_)_2_	*F*(000) = 1296
*M**_r_* = 656.27	*D*_x_ = 2.040 Mg m^−^^3^
Monoclinic, *C*2/*c*	Mo *K*α radiation, λ = 0.71073 Å
Hall symbol: -C 2yc	Cell parameters from 5781 reflections
*a* = 14.612 (4) Å	θ = 2.3–28.3°
*b* = 12.498 (4) Å	µ = 3.03 mm^−^^1^
*c* = 13.342 (4) Å	*T* = 293 K
β = 118.728 (4)°	Block, colourless
*V* = 2136.6 (11) Å^3^	0.35 × 0.32 × 0.28 mm
*Z* = 4	

### Data collection

**Table:**

Bruker APEXII CCD diffractometer	1881 independent reflections
Radiation source: fine-focus sealed tube	1847 reflections with *I* > 2σ(*I*)
graphite	*R*_int_ = 0.017
φ and ω scans	θ_max_ = 25.0°, θ_min_ = 2.3°
Absorption correction: multi-scan *SADABS* (Bruker, 1997)	*h* = −13→17
*T*_min_ = 0.417, *T*_max_ = 0.484	*k* = −14→12
5224 measured reflections	*l* = −15→15

### Refinement

**Table:**

Refinement on *F*^2^	Primary atom site location: structure-invariant direct methods
Least-squares matrix: full	Secondary atom site location: difference Fourier map
*R*[*F*^2^ > 2σ(*F*^2^)] = 0.015	Hydrogen site location: inferred from neighbouring sites
*wR*(*F*^2^) = 0.038	H atoms treated by a mixture of independent and constrained refinement
*S* = 1.02	*w* = 1/[σ^2^(*F*_o_^2^) + (0.0211*P*)^2^ + 2.5852*P*] where *P* = (*F*_o_^2^ + 2*F*_c_^2^)/3
1881 reflections	(Δ/σ)_max_ = 0.001
180 parameters	Δρ_max_ = 0.58 e Å^−^^3^
8 restraints	Δρ_min_ = −0.62 e Å^−^^3^

### Special details

**Table:**

Geometry. All e.s.d.'s (except the e.s.d. in the dihedral angle between two l.s. planes) are estimated using the full covariance matrix. The cell e.s.d.'s are taken into account individually in the estimation of e.s.d.'s in distances, angles and torsion angles; correlations between e.s.d.'s in cell parameters are only used when they are defined by crystal symmetry. An approximate (isotropic) treatment of cell e.s.d.'s is used for estimating e.s.d.'s involving l.s. planes.

### Fractional atomic coordinates and isotropic or equivalent isotropic displacement parameters (Å^2^)

**Table:**

	*x*	*y*	*z*	*U*_iso_*/*U*_eq_	
Eu1	0.0000	0.184699 (10)	0.2500	0.02398 (6)	
O1	0.27428 (13)	0.30594 (12)	0.37370 (16)	0.0421 (4)	
O1W	−0.17399 (12)	0.12138 (14)	0.15960 (17)	0.0453 (4)	
H1WB	−0.199 (2)	0.0622 (15)	0.144 (2)	0.044 (8)*	
H1WA	−0.2202 (13)	0.1684 (17)	0.134 (3)	0.063 (10)*	
O2	0.10558 (12)	0.33068 (12)	0.25718 (14)	0.0350 (4)	
O2W	−0.04309 (13)	0.22924 (14)	0.05884 (14)	0.0390 (4)	
H2WB	−0.0982 (17)	0.213 (2)	0.002 (2)	0.051 (8)*	
H2WA	−0.021 (2)	0.2840 (19)	0.045 (3)	0.061 (10)*	
O3	0.00037 (14)	0.00420 (15)	0.33097 (17)	0.0495 (4)	
O4	0.0000	−0.1458 (2)	0.2500	0.1018 (15)	
O5	0.08162 (14)	0.35758 (14)	−0.01819 (16)	0.0462 (4)	
O6	0.11039 (14)	0.52783 (13)	−0.00657 (16)	0.0471 (4)	
O7	0.23063 (12)	0.42067 (12)	0.10876 (14)	0.0386 (4)	
N1	0.23834 (19)	0.69898 (16)	0.3531 (2)	0.0390 (5)	
N2	0.0000	−0.0487 (2)	0.2500	0.0506 (9)	
N3	0.13887 (14)	0.43631 (14)	0.02637 (16)	0.0312 (4)	
C1	0.3165 (2)	0.63532 (19)	0.4224 (2)	0.0403 (5)	
H1	0.3784	0.6647	0.4788	0.048*	
C2	0.30538 (18)	0.52613 (18)	0.4104 (2)	0.0353 (5)	
H2	0.3604	0.4813	0.4567	0.042*	
C3	0.21208 (16)	0.48366 (16)	0.32907 (17)	0.0262 (4)	
C4	0.13218 (17)	0.55193 (17)	0.25749 (19)	0.0317 (5)	
H4	0.0691	0.5246	0.2012	0.038*	
C5	0.1479 (2)	0.65997 (19)	0.2713 (2)	0.0387 (5)	
H5	0.0954	0.7066	0.2235	0.046*	
C6	0.19639 (16)	0.36402 (16)	0.31971 (17)	0.0270 (4)	
H6	0.248 (2)	0.773 (3)	0.367 (2)	0.050 (8)*	

### Atomic displacement parameters (Å^2^)

**Table:**

	*U*^11^	*U*^22^	*U*^33^	*U*^12^	*U*^13^	*U*^23^
Eu1	0.01991 (9)	0.01759 (9)	0.02573 (9)	0.000	0.00400 (6)	0.000
O1	0.0282 (8)	0.0270 (8)	0.0463 (10)	0.0018 (6)	−0.0019 (8)	0.0010 (7)
O1W	0.0250 (8)	0.0221 (9)	0.0734 (13)	−0.0035 (7)	0.0112 (8)	−0.0035 (8)
O2	0.0258 (8)	0.0258 (8)	0.0379 (9)	−0.0057 (6)	0.0028 (7)	0.0032 (6)
O2W	0.0332 (9)	0.0382 (10)	0.0296 (8)	−0.0127 (8)	0.0023 (7)	0.0047 (7)
O3	0.0463 (10)	0.0388 (10)	0.0516 (11)	−0.0004 (8)	0.0140 (9)	0.0148 (8)
O4	0.088 (3)	0.0177 (14)	0.202 (5)	0.000	0.071 (3)	0.000
O5	0.0419 (10)	0.0339 (9)	0.0486 (10)	−0.0067 (8)	0.0104 (8)	−0.0042 (8)
O6	0.0487 (10)	0.0281 (9)	0.0581 (11)	0.0106 (8)	0.0207 (9)	0.0114 (8)
O7	0.0331 (8)	0.0291 (8)	0.0428 (9)	0.0014 (6)	0.0098 (7)	0.0034 (7)
N1	0.0544 (13)	0.0233 (10)	0.0466 (12)	−0.0076 (9)	0.0302 (11)	−0.0047 (8)
N2	0.0305 (15)	0.0215 (14)	0.083 (3)	0.000	0.0135 (16)	0.000
N3	0.0333 (10)	0.0281 (10)	0.0337 (10)	0.0021 (8)	0.0172 (8)	0.0016 (8)
C1	0.0452 (14)	0.0326 (13)	0.0373 (13)	−0.0140 (11)	0.0151 (11)	−0.0061 (10)
C2	0.0327 (11)	0.0286 (12)	0.0336 (12)	−0.0056 (9)	0.0070 (10)	0.0001 (9)
C3	0.0277 (10)	0.0254 (10)	0.0253 (10)	−0.0023 (8)	0.0127 (9)	−0.0003 (8)
C4	0.0275 (10)	0.0288 (11)	0.0359 (12)	0.0009 (9)	0.0127 (9)	0.0017 (9)
C5	0.0438 (14)	0.0271 (11)	0.0479 (14)	0.0064 (10)	0.0242 (12)	0.0051 (10)
C6	0.0255 (10)	0.0237 (11)	0.0239 (10)	−0.0034 (8)	0.0056 (8)	−0.0015 (8)

### Geometric parameters (Å, °)

**Table:**

Eu1—O2	2.3614 (16)	O5—N3	1.242 (3)
Eu1—O2^i^	2.3614 (16)	O6—N3	1.224 (2)
Eu1—O1W	2.3657 (17)	O7—N3	1.276 (2)
Eu1—O1W^i^	2.3657 (17)	N1—C1	1.333 (4)
Eu1—O2W^i^	2.3806 (18)	N1—C5	1.337 (4)
Eu1—O2W	2.3806 (18)	N1—H6	0.94 (3)
Eu1—O3^i^	2.5000 (19)	N2—O3^i^	1.264 (2)
Eu1—O3	2.5000 (19)	C1—C2	1.375 (3)
Eu1—N2	2.917 (3)	C1—H1	0.9300
O1—C6	1.246 (3)	C2—C3	1.377 (3)
O1W—H1WB	0.805 (17)	C2—H2	0.9300
O1W—H1WA	0.834 (16)	C3—C4	1.390 (3)
O2—C6	1.251 (3)	C3—C6	1.509 (3)
O2W—H2WB	0.826 (17)	C4—C5	1.367 (3)
O2W—H2WA	0.818 (18)	C4—H4	0.9300
O3—N2	1.264 (2)	C5—H5	0.9300
O4—N2	1.214 (4)		
			
O2—Eu1—O2^i^	78.82 (8)	Eu1—O1W—H1WA	115.7 (15)
O2—Eu1—O1W	143.15 (6)	H1WB—O1W—H1WA	111 (2)
O2^i^—Eu1—O1W	73.54 (6)	C6—O2—Eu1	138.33 (14)
O2—Eu1—O1W^i^	73.54 (6)	Eu1—O2W—H2WB	125 (2)
O2^i^—Eu1—O1W^i^	143.15 (6)	Eu1—O2W—H2WA	121 (2)
O1W—Eu1—O1W^i^	140.91 (8)	H2WB—O2W—H2WA	109 (3)
O2—Eu1—O2W^i^	86.83 (6)	N2—O3—Eu1	95.99 (15)
O2^i^—Eu1—O2W^i^	72.18 (6)	C1—N1—C5	122.0 (2)
O1W—Eu1—O2W^i^	106.89 (7)	C1—N1—H6	116.8 (18)
O1W^i^—Eu1—O2W^i^	82.30 (7)	C5—N1—H6	121.2 (18)
O2—Eu1—O2W	72.18 (6)	O4—N2—O3	121.53 (14)
O2^i^—Eu1—O2W	86.83 (6)	O4—N2—O3^i^	121.53 (14)
O1W—Eu1—O2W	82.30 (7)	O3—N2—O3^i^	116.9 (3)
O1W^i^—Eu1—O2W	106.89 (7)	O4—N2—Eu1	180.0
O2W^i^—Eu1—O2W	152.96 (9)	O3—N2—Eu1	58.47 (14)
O2—Eu1—O3^i^	125.48 (7)	O3^i^—N2—Eu1	58.47 (14)
O2^i^—Eu1—O3^i^	144.49 (6)	O6—N3—O5	122.3 (2)
O1W—Eu1—O3^i^	72.50 (6)	O6—N3—O7	119.18 (19)
O1W^i^—Eu1—O3^i^	72.37 (6)	O5—N3—O7	118.56 (18)
O2W^i^—Eu1—O3^i^	128.20 (6)	N1—C1—C2	119.9 (2)
O2W—Eu1—O3^i^	78.67 (7)	N1—C1—H1	120.1
O2—Eu1—O3	144.49 (6)	C2—C1—H1	120.1
O2^i^—Eu1—O3	125.48 (7)	C1—C2—C3	119.4 (2)
O1W—Eu1—O3	72.37 (6)	C1—C2—H2	120.3
O1W^i^—Eu1—O3	72.50 (6)	C3—C2—H2	120.3
O2W^i^—Eu1—O3	78.67 (7)	C2—C3—C4	119.4 (2)
O2W—Eu1—O3	128.20 (6)	C2—C3—C6	120.06 (19)
O3^i^—Eu1—O3	51.07 (10)	C4—C3—C6	120.50 (19)
O2—Eu1—N2	140.59 (4)	C5—C4—C3	118.8 (2)
O2^i^—Eu1—N2	140.59 (4)	C5—C4—H4	120.6
O1W—Eu1—N2	70.46 (4)	C3—C4—H4	120.6
O1W^i^—Eu1—N2	70.46 (4)	N1—C5—C4	120.4 (2)
O2W^i^—Eu1—N2	103.52 (4)	N1—C5—H5	119.8
O2W—Eu1—N2	103.52 (4)	C4—C5—H5	119.8
O3^i^—Eu1—N2	25.54 (5)	O1—C6—O2	124.92 (19)
O3—Eu1—N2	25.54 (5)	O1—C6—C3	117.98 (18)
Eu1—O1W—H1WB	133 (2)	O2—C6—C3	117.09 (18)

Symmetry codes: (i) −*x*, *y*, −*z*+1/2.

### Hydrogen-bond geometry (Å, °)

**Table:**

*D*—H···*A*	*D*—H	H···*A*	*D*···*A*	*D*—H···*A*
O1W—H1WB···O7^ii^	0.81 (2)	1.99 (2)	2.790 (2)	175 (3)
O1W—H1WA···O1^i^	0.83 (2)	1.87 (2)	2.653 (2)	155 (2)
O2W—H2WB···O1^iii^	0.83 (2)	1.84 (2)	2.661 (2)	173 (3)
O2W—H2WA···O5	0.82 (2)	2.23 (2)	2.958 (3)	148 (3)
N1—H6···O7^iv^	0.94 (3)	1.88 (3)	2.814 (3)	179 (3)

Symmetry codes: (ii) *x*−1/2, *y*−1/2, *z*; (i) −*x*, *y*, −*z*+1/2; (iii) *x*−1/2, −*y*+1/2, *z*−1/2; (iv) −*x*+1/2, *y*+1/2, −*z*+1/2.
